# Illuminating the Prevalence of *Trypanosoma brucei s*.*l*. in *Glossina* Using LAMP as a Tool for Xenomonitoring

**DOI:** 10.1371/journal.pntd.0004441

**Published:** 2016-02-18

**Authors:** Lucas J. Cunningham, Jessica K. Lingley, Lee R. Haines, Joseph M. Ndung’u, Stephen J. Torr, Emily R. Adams

**Affiliations:** 1 Vector Biology, Liverpool School of Tropical Medicine, Liverpool, United Kingdom; 2 Neglected Tropical Disease Programme, Foundation for Innovation New Diagnostics (FIND), Geneva, Switzerland; 3 Parasitology, Liverpool School of Tropical Medicine, Liverpool, United Kingdom; 4 Warwick Medical School, University of Warwick, Coventry, United Kingdom; Institute of Tropical Medicine Antwerp, BELGIUM

## Abstract

**Background:**

As the reality of eliminating human African trypanosomiasis (HAT) by 2020 draws closer, the need to detect and identify the remaining areas of transmission increases. Here, we have explored the feasibility of using commercially available LAMP kits, designed to detect the *Trypanozoon* group of trypanosomes, as a xenomonitoring tool to screen tsetse flies for trypanosomes to be used in future epidemiological surveys.

**Methods and Findings:**

The DNA extraction method was simplified and worked with the LAMP kits to detect a single positive fly when pooled with 19 negative flies, and the absolute lowest limit of detection that the kits were able to work at was the equivalent of 0.1 trypanosome per ml. The DNA from *Trypanosoma brucei brucei* could be detected six days after the fly had taken a blood meal containing dead trypanosomes, and when confronted with a range of non-target species, from both laboratory-reared flies and wild-caught flies, the kits showed no evidence of cross-reacting.

**Conclusion:**

We have shown that it is possible to use a simplified DNA extraction method in conjunction with the pooling of tsetse flies to decrease the time it would take to screen large numbers of flies for the presence of *Trypanozoon* trypanosomes. The use of commercially-available LAMP kits provides a reliable and highly sensitive tool for xenomonitoring and identifying potential sleeping sickness transmission sites.

## Author Summary

Recent control efforts have reduced the global incidence of *Gambiense* human African trypanosomiasis (HAT) to <5,000 cases per year, strengthening the prospect of eliminating the disease as a public health problem by 2020. To meet this goal, new methods for identifying transmission must be explored to provide a cost-effective way of identifying hotspots and areas of re-emergence; commercial loop-mediated isothermal amplification (LAMP) kits that detect the trypanosome subgenus, responsible for the two forms of sleeping sickness, have been developed. The LAMP kits were tested to assess their sensitivity, specificity and suitability as a method of screening the vector of the disease, *Glossina*, for *Trypanozoon* infection, in xenomonitoring campaigns. A simplified DNA extraction process that worked in conjunction with the LAMP kits on pooled samples demonstrated a faster method of processing large numbers of flies compared to other molecular tools. The kits performed well in our experiments and demonstrated the ability of detecting low levels of target DNA, equivalent to 0.1 trypanosome per ml. The lack of cross reaction with non-target species of trypanosomes makes the kits reliable in so far as they will only react with the *Trypanozoon* group of parasites of which the two human forms of the disease belong, however, further species-specific tests would need to be undertaken to identify HAT areas on selected samples.

## Introduction

Human African trypanosomiasis (HAT), commonly known as sleeping sickness, is a parasitic disease caused by two sub-species of trypanosome, *Trypanosoma brucei gambiense* in West and Central Africa, which causes a chronic disease and *T*.*b*. *rhodesiense* in East and southern Africa which causes an acute disease. These pathogens are transmitted by several species of tsetse flies (*Glossina sp*.). Through active case detection and treatment, cases of *Gambiense* sleeping sickness are estimated to be ≤5000 as of 2014 [1]. The greatest contribution to overall HAT burden is the west African (*Gambiense*) form of the disease as this accounts for 98% of all sleeping sickness cases in most recent estimates[2]. This is reflected by the number of people at risk of the disease, with 57 million people at risk of *Gambiense* sleeping sickness compared to 12.3 million at risk of the *Rhodesiense* form [3]. The methods of controlling HAT differ according to the underlying nature of the two forms, with more emphasis being placed on vector control in the case of *Rhodesiense* HAT.

*Gambiense* HAT is generally considered to be anthroponotic and control is achieved mainly by screening and treating human cases; vector control has not played an important role. However with the development of more cost-effective methods [4] it seems likely that tsetse control will play an important role in efforts against *Gambiense* HAT. [5].

Detection of *Gambiense* HAT relies largely on active or passive screening of the population at risk. Active screening is difficult; screening >70% of a target population is seldom achieved [6] and there are sensitivity issues as the card agglutination test (CATT) is not 100% accurate and is often one of a series of tests including lymph node puncture, fresh blood and thick blood film examination [7–9]. Moreover, as the prevalence of HAT decreases, the cost of detecting each case increases, leading to active screening programmes being scaled down or abandoned as other health priorities take precedent [10, 11].

An alternative to screening people is to screen vectors for the presence of the pathogen. This approach, termed xenomonitoring, has proven useful in the control of other vector transmitted diseases such as lymphatic filariasis [12]. Rather than screening people, tsetse flies might be caught using simple traps and subsequently analysed for presence of *T*. *brucei s*.*l*. By monitoring trypanosomes in tsetse, xenomonitoring would measure current rates of transmission in a defined area. By contrast, the chronic nature of *Gambiense* HAT and the mobility of humans mean that cases are detected months, if not years, after infection, and in places far removed from sites of transmission.

Hitherto, detection of pathogenic trypanosomes in individual tsetse flies relied largely on dissection and microscopic evaluation of individual flies which is technically demanding and time consuming [13]. Molecular techniques are more sensitive than classical dissection, but they are also laborious, requiring skilled workers and well-equipped laboratories [14]. Xenomonitoring for HAT requires novel methods that are easy, rapid, cheap, specific and sensitive. A method that offers the prospect of being fit for this purpose is coupling a rapid and robust method of extracting trypanosome DNA from a fly with a simplified molecular test such as loop-mediated isothermal amplification (LAMP). The LAMP test for HAT amplifies the repetitive insertion mobile element (RIME) of the *Trypanozoon* group [15]. LAMP is carried out at a constant temperature, thereby removing the need for thermocyclers, and results are read visually [16].

Studies of trypanosome infection in tsetse flies are generally concerned with quantifying the prevalence of pathogens in the vector population. For this purpose, the status of infection in individual flies must be quantified; only tsetse with a mature infection where *T*. *brucei s*.*l*. are observed in the salivary glands are infectious. For xenomonitioring however, the aim is to detect the presence or absence of pathogens in a vector population. This specific aim offers two opportunities to improve the cost-effectiveness of screening. First, pooled groups rather than single individuals might be screened for the presence of pathogens. Second, a molecular method may be able to detect trypanosomes that have been recently ingested but which will not lead to a mature infection. Older tsetse flies are much less susceptible to infection with *T*. *brucei* [17]. However, flies that are refractory to infection will nonetheless ingest trypanosomes from infected hosts and a sensitive method may be able to detect these transient trypanosomes in recently-fed flies.

Previous work has demonstrated the ability of an in-house LAMP assay to detect *T*. *brucei* in laboratory-infected tsetse and in pooled groups of tsetse [18]. Here, we assessed various simple methods for (i) extracting trypanosome DNA from tsetse, and (ii) the potential of using a commercially-available Loopamp *Trypanosoma brucei* detection kit developed by Eiken Chemical Co (Japan). Performance of LAMP was assessed with laboratory flies experimentally infected with trypanosomes and wild-caught tsetse flies from an area of northern Uganda where *T*. *brucei s*.*l*. is present.

## Methods

Tsetse fly samples were tested using the Loopamp kit to determine the presence of trypanosome DNA, and the limit of detection of LAMP. DNA extraction methods were optimised for field settings and tested with the LAMP kits in pooled fly assays. The specificity of the kit was tested against flies infected with non-target species of trypanosomes, most likely *Trypanosoma congolense sl*. and *Trypanosoma vivax* in both laboratory-infected (*Glossina morsitans morsitans*) and wild flies (*Glossina fuscipes fuscipes*).

### DNA Extraction

Tsetse flies were dissected and their midguts stored in 60μl of 100% ethanol (EtOH). At the Liverpool School of Tropical Medicine (LSTM) a standard DNA extraction procedure, hereafter referred to as the Chelex method, was followed: 70ul of distilled water was added to the midgut sample followed by centrifugation at 13,000rpm (15sec) and removal of 100μl of supernatant. Tissue samples were washed three times by adding and removing 100μl of distilled water. To the washed tissue a 100μl suspension of Chelex and Proteinase K (20mg/ml) was added to give a final concentration of 1% Chelex, and incubated at 56°C for 1 h. The sample was then incubated for 30 minutes at 93°C, centrifuged at 13,000rpm (15sec), the supernatant removed, and stored at -20C.

### Simplification of DNA Extraction

The current Chelex DNA extraction method, although robust, is not field-friendly and takes ~2 h to complete with a number of steps and components, some of which require a cold chain. To use this kit in the field, the current method needs to be optimised to ensure it is rapid, robust and simple. Five alternative DNA extraction methods aimed at reducing the time and complexity of the current Chelex extraction method for use in field studies were designed (Table 1).

**Table 1:** Details of five alternative field-friendly DNA extraction methods.

Method	Spin 13,000 rpm	Wash x3	Spin 13,000 rpm	5% Chelex or 1% TE	Proteinase K	Incubation		Total Time (Min)
						56°C	93°C	
**Chelex**	Y	Y	Y	Chelex	Y	60 m	30 m	120
**1/2 time chelex**	Y	Y	Y	Chelex	Y	30 m	15 m	50
**1/4 time chelex**	Y	Y	Y	Chelex	Y	15 m	7.5 m	30
**TE wash off alcohol**	Y	Y	Y	TE			15 m	45
**TE with chelex**	Y	*		Chelex			15 m	15
**TE leave in alcohol**	Y			TE			15 m	22

*The alcohol was not washed off, instead the samples were transferred out of the EtOH and into a clean empty tube

These different DNA extraction methods were tested against two sets of trypanosome concentrations at 10^2^ and 10^4^ trypanosomes per ml, with each of these concentrations being tested in eight replicates with a combined total of 16 replicates across the two concentrations.

### LAMP

The RIME LAMP test was performed using the Loopamp *Trypanosoma brucei* Detection Kits manufactured by Eiken Chemical Co.,Ltd, Japan, according to the manufacturer’s specifications. Briefly, 2.5μl of template DNA was added to 22.5 μl of nuclease-free water. LAMP reagents were reconstituted in the lids of the tubes, and after two minutes incubation, the tubes were inverted five times. A thermocycler was used to heat the samples for forty minutes at 65°C and then at 85°C for five minutes to stop the reaction. Results were determined by visualising presence or absence of fluorescence in the reaction tubes on a UV gel imager, and each sample was classed as positive or negative based on two separate blind screenings by two of the co-authors (LJC, JKL).

### PCR

Alongside the RIME LAMP assays, samples were also amplified using TBR primers[19] that target the same gene as those designed by Moser[20] but are situated inside Moser’s primers, thus producing a smaller product of 117bp compared with the original product of 177bp and a nested universal trypanosome ITS primer set[21] (Table 2).

**Table 2:** Primers used in this study.

Primer name	Target species	Primer sequence 5’-3’	Published/designed by
TBR Forward	*T*. *brucei s*.*l*.	TGCGCAGTTAACGCTATTATACA	Kazibwe 2008
TBR Reverse	*T*. *brucei s*.*l*.	AAAGAACAGCGTTGCAAACTT	Kazibwe 2008
Tryp 1	*Trypanosomatidae*	AAGCCAAGTCATCCATCG	Adams et al. 2006
Tryp 2	*Trypanosomatidae*	TAGAGGAAGCAAAAG	Adams et al. 2006
Tryp 3	*Trypanosomatidae*	TGCAATTATTGGTCGCGC	Adams et al. 2006
Tryp 4	*Trypanosomatidae*	CTTTGCTGCGTTCTT	Adams et al. 2006

The PCR reactions consisted of 12.5 μl MyTaq Red Mix (Bioline), 1 μl of forward and reverse primer (25mmol), 8.5 μl of nuclease free water and 2 μl of DNA template. For the TBR primers the PCR cycles were: an initial denaturation step at 93°C for 2 mins, followed by 35 cycles at 94°C for 10 s, 55°C for 10 s and 72°C for 10 s with a final extension step of 30 s at 72°C, for the nested PCR 1 μl of PCR product from the first nest was added to the second to act as the template.

For both nests of the ITS primers the PCR cycle had an initial denaturation at 95°C for 5 mins followed by 35 cycles of 94°C for 15 s, 54°C for 15 s and 72°C for 10 s with a final extension step at 72°C for 5 mins.

### Limit of Detection

Colony-reared *G*. *m*. *morsitans* were dissected three days after their first blood meal, the midguts were collected into individual tubes and preserved in 100% EtOH. A trypanosome dilution series was created by first making a stock concentration of 2x10^5^ trypanosomes per ml using a haemocytometer (20). The trypanosomes were then heated at 93°C for 30 mins to lyse the parasites and extract the DNA. Using the eluted DNA, a tenfold dilution series was then created with the equivalent number of parasites for each gradient step as follows:

2x10^5^/ml (stock), 2x10^4^/ml, 2x10^3^/ml, 2x10^2^/ml, 2x10^1^/ml, 2/ml, 0.2/ml and 0.02/ml.

To prepare the samples for analysis a modified version of the Chelex method was used. To remove residual EtOH from the preserved tsetse tissue, midguts were washed three times as previously described. To each washed midgut, 50μl of a trypanosome DNA concentration was added. Following the addition of trypanosome DNA, 50μl of a Chelex suspension (10% Chelex with a 2% proteinase K concentration) was added to the midgut tissue for a final volume of 100μl. The DNA concentration series represents 1x10^5^/ml, 1x10^4^/ml, 1x10^3^/ml, 1x10^2^/ml, 1x10^1^/ml, 1/ml, 0.1/ml, 0.01/ml trypanosomes suspended in 5% Chelex suspension/1% proteinase K. Each DNA dilution was processed according to the standard Chelex extraction method. Table 3 summarises the number of trypanosomes in the DNA dilution series per ml, per 100μl, and per reaction for both the LAMP (2.5μl of template) and PCR (2μl of template) assays.

**Table 3:** A breakdown explaining the equivalent number of trypanosomes at different volumes used in the study.

	Equivalent number of trypanosomes present per DNA concentration at:							
	1x10^5/ml	1x10^4/ml	1x10^3/ml	1x10^2/ml	1x10^1/ml	1/ml	0.1/ml	0.01/ml
**Trypanosomes ml**	100000	10000	1000	100	10	1	0.1	0.01
**per 100μl sample**	10000	1000	100	10	1	0.1	0.01	0.001
**per LAMP assay**	250	25	2.5	0.25	0.025	0.0025	0.00025	0.000025
**ng/μl LAMP assay**	2.5x10^-2^	2.5x10^-3^	2.5x10^-4^	2.5x10^-5^	2.5x10^-6^	2.5x10^-7^	2.5x10^-8^	2.5x10^-9^
**per PCR assay**	200	20	2	0.2	0.02	0.002	0.0002	0.00002
**ng/μl TBR assay**	2x10^-2^	2x10^-3^	2x10^-4^	2x10^-5^	2x10^-6^	2x10^-7^	2x10^-8^	2x10^-9^

### Pooling

Infected *G*.*m*. *morsitans* flies were generated by adding 200μl of thawed *T*.*b*. *brucei* GFP J10 blood stabilates to five ml of defibrinated horse blood (TCS Biosciences Ltd). Teneral flies were fed through a silicon membrane placed over the blood which was heated to 37°C. After seven days the flies were dissected and their midguts visually screened for infection using a compound microscope. Positive midguts were then stored individually in 100% EtOH and later they were processed using the chosen simplified DNA extraction method. Parallel to the infected flies a group of uninfected flies were also generated apart from the omission of the 200μl of *T*. *b*. *brucei* blood stabilate to the five ml of horse blood. The midguts of negative flies were screened prior to being stored in the EtOH with microscopy and also later, after DNA extraction using the Chelex method, with TBR PCR to ensure they were truly uninfected.

Pools of tsetse flies were generated by adding 3μl of DNA template from a single positive fly sample to pools of four, nine and nineteen uninfected fly samples, in which each fly contributed 3μl of DNA extract to the pool. A total of 9 replicates were performed per pool and each pool was tested with LAMP.

### Persistence of DNA in Tsetse Material

To assess the ability of the Loopamp kit to detect trypanosomes from a previous blood meal 200μl of a 10^6^/ml *T*. *b*. *brucei* blood stabilate was incubated for 15 minutes at 54°C to kill the trypanosomes and hence prevent establishment of an infection in the tsetse. The heat-killed trypanosomes were added to 5ml of defibrinated horse blood. The viability of the parasites was determined through microscopy and the use of the stain Trypan blue[22]. If no living trypanosomes were observed the blood was then fed to teneral flies. A similar volume of uninfected blood was heated and fed to a control group of flies. Every 24 hours after the initial blood meal, three experimental flies and one control fly were dissected and the midgut removed and stored in 95% EtOH. During each dissection the midgut samples were screened visually for living trypanosomes using a compound microscope as a secondary measure to ensure no trypanosomes had survived and had started to infect the flies.

The midgut samples were processed using the Chelex method followed by LAMP and PCR assays as described. The experiments were repeated until the LAMP and PCR tests showed two consecutive days of negative results in all experimental fly samples.

### Mixed Infections

LAMP kits were tested for cross reactivity with single and mixed species infections of both *T*. *congolense* (1/148) and *T*.*b*. *brucei* (GFP J10). Flies were infected by artificial membrane feeding [23]. Partially fed flies were removed. On the seventh day post-infection all flies were dissected and their midguts screened for trypanosomes by microscopy [24].

The four groups were processed using the Chelex method and analysed using the *Trypanozoon* specific TBR primers and the universal ITS primers alongside the RIME LAMP kits.

### Wild-Caught Tsetse Flies

From NW Uganda, 449 *G*.*f*. *fuscipes* were caught and screened by microscopy, universal ITS primers and the LAMP kits. The flies were caught from April to June 2013 from eight trap sites (Northings 381161–383674, Eastings 276506–287545) in the district of Koboko. The flies were dissected in the field and their mouthparts, salivary glands and midguts were separately screened for trypanosomes by microscopy. All samples were then stored individually in 100% EtOH and shipped at room temperature to LSTM where they underwent the Chelex extraction prior to PCR and LAMP analysis.

## Results

### Simplification of DNA Extraction

The results for the six different DNA extraction methods, tested with the LAMP kits, are shown in Table 4. The method that produced the highest number of positive results was ‘½ time Chelex ‘followed by ‘TE with Chelex’ detecting 13 and 12 positive samples respectively out of a total of 16 positives. The lowest scoring methods were ‘TE Leave in alcohol’ and ‘TE wash off alcohol’. Taking into account the number of steps involved for each method and the total time it takes ‘TE with Chelex’ was deemed the most efficient as it took half the time of ‘½ Chelex’ but only identified one less positive sample (Table 4).

**Table 4:** Overview of the results for the different DNA extraction methods.

DNA Extraction methods	Positive results (trypanosome/ml)		Total number of positives	% of positive samples identified
	10^2	10^4		
Chelex	4	7	11	69
½ time Chelex	6	7	13	81
¼ time Chelex	5	6	11	69
TE Leave in alcohol	2	5	7	44
TE wash off alcohol	1	8	9	56
TE with Chelex	8	4	12	75

### Limit of Detection

**Fig 1:**
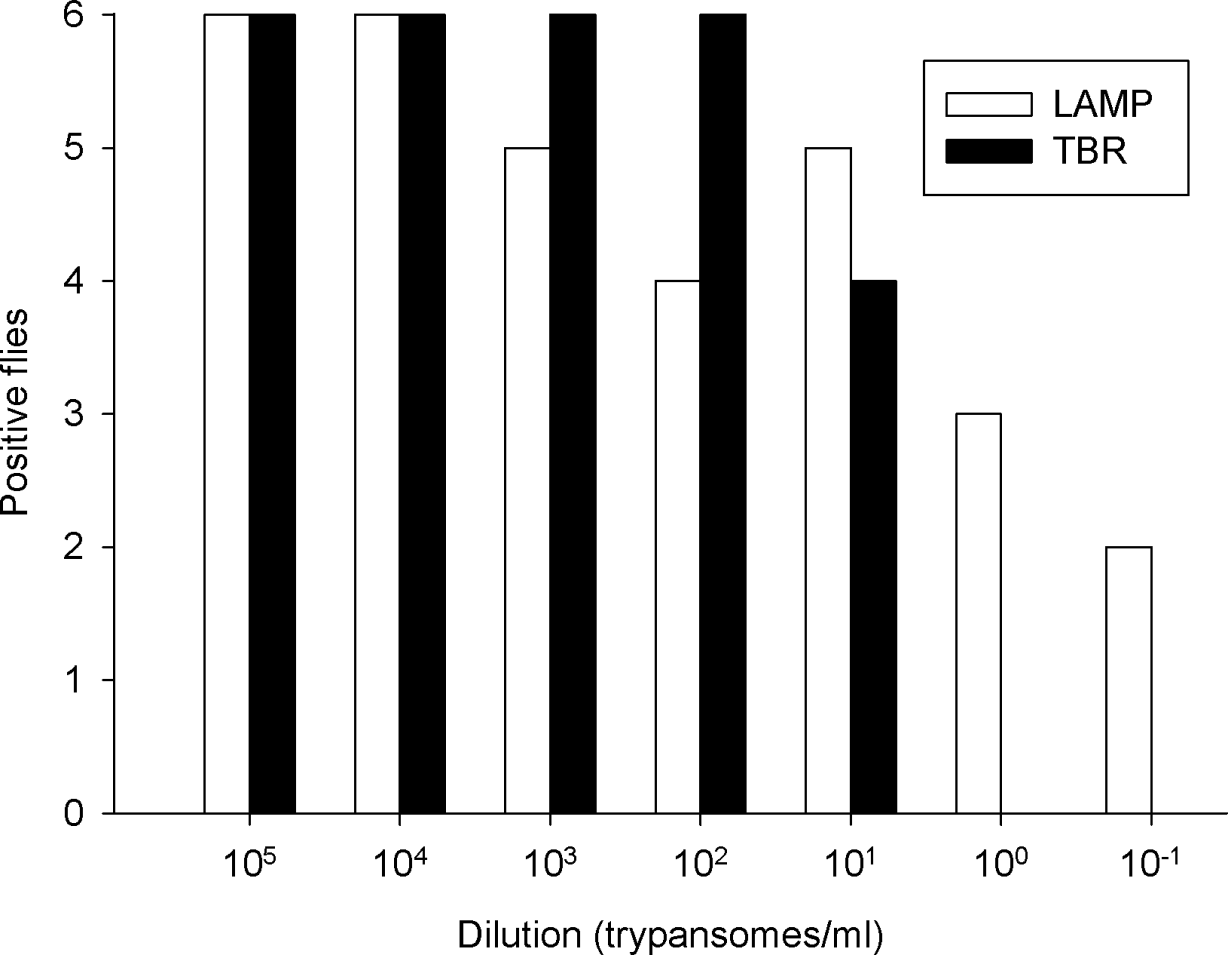
Limit of detection results for LAMP and TBR primers, a total of six flies were used for each dilution gradient for both assays.

The results (Fig 1) shows that the LAMP kit was able to detect 100% of the spiked samples up until 10^4^ trypanosomes per ml, after which there was a decline in the ability to detect the trypanosome DNA until 10^−1^ trypanosomes per ml. The sensitivity of the LAMP kit when compared with the TBR PCR, at the extreme limit of detection, was better by a factor of two as the TBR PCR had a limit of detection of 10^1^ trypanosomes/ml. Although the template volumes for the LAMP and TBR assays varied slightly by 0.5 μl (or 25%) this variation is too small to explain the two fold, (or 100 x) greater difference in the sensitivity of the two assays.

### Pooling

Pooling experiments show that LAMP was able to amplify one positive midgut in pools of four, nine and 19 uninfected midguts—this was repeated in nine independent tests. There were two kit failures, one in ratio 1:5 and the other in 1:20. The failure was caused by the dried reagents failing to properly dissolve and integrate into the reaction.

### DNA Persistence

**Fig 2:**
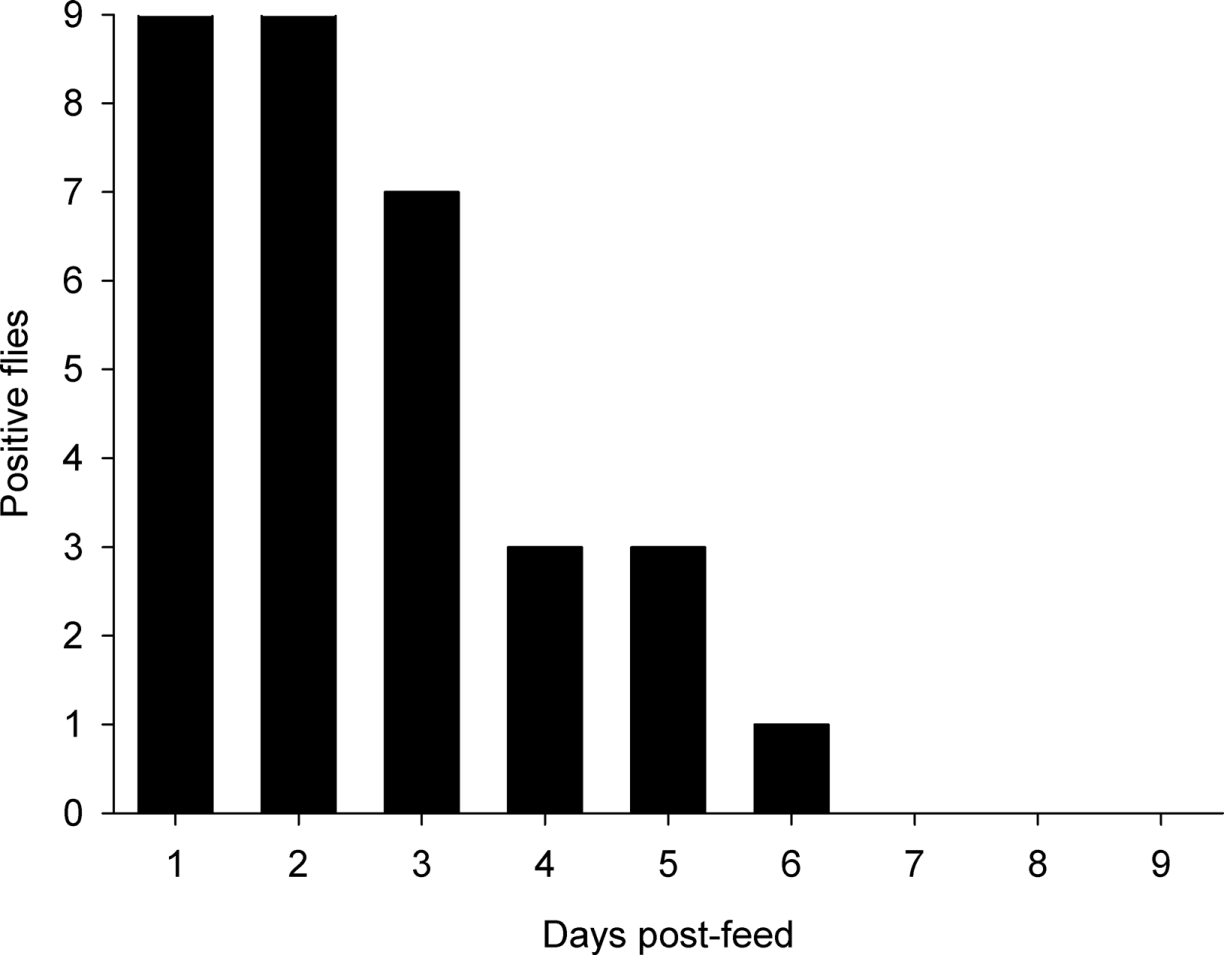
Persistence of *T*. *b*. *brucei* DNA in *G*. *m*. *morsitans*.

The number of tsetse that were able to give a positive LAMP results declined from 100% after 48 hours to just 11% by day 6 (Fig 2).

### Mixed Infections

Thirteen out of sixteen surviving flies from a mono *T*.*b*. *brucei* infection were identified as positive by both microscopy and TBR PCR (Table 5). When the same samples were tested with LAMP, all 16 flies tested positive. Of the 17 surviving flies from the *T*. *congolense* single infection, 14 were positive by microscopy, 13 tested positive with the ITS primers (which amplifies and differentiates both *T*. *brucei and T*. *congolense)* and none were detected by LAMP, which is specific for *T*. *brucei s*.*l*. Of the mixed infection group, 15 flies were positive by microscopy, 18 tested positive using the generic ITS primers, 17 tested positive with the *brucei*-specific TBR primers and 20 tested positive with the LAMP kits. Of the 18 flies identified as positive with the ITS PCR, one had a single *T*. *congolense* infection, 3 had single *T*. *b*. *brucei* infections and 14 had mixed infections. Both the TBR PCR and LAMP did not cross-react with the single *T*. *congolense* infection (Table 5). The negative control group tested negative with microscopy, PCR and LAMP assays. LAMP is shown to be more sensitive than either the ITS or TBR primer sets.

**Table 5:** The results of testing cross reactivity with flies containing single and mixed infections of *T*.*b*. *brucei* and *T*. *congolense*.

Infection	Assay	Positive	Negative	Lost Flies	Total
**Single *T*. *brucei* infection**	LAMP	16	0	9	25
	TBR	13	3	9	25
	Microscopy	13	3	9	25
**Single *T*. *congolense* infection**	LAMP	0	17	8	25
	ITS	13	4	8	25
	Microscopy	14	3	8	25
**Mixed infection *T*. *b*. *brucei* and *T*. *congolense***	LAMP	20	3	2	25
	TBR	17	6	2	25
	ITS	18	5	2	25
	Microscopy	15	8	2	25
**Negative control**	LAMP	0	16	9	25
	TBR	0	16	9	25
	ITS	0	16	9	25
	Microscopy	0	16	9	25

### Wild Flies

Of the 449 wild-caught tsetse, microscopy identified one salivary gland positive *T*. *brucei s*.*l*. sample, the universal ITS primers identified two *T*. *brucei* sl. samples while LAMP identified six. The ITS assay identified 40 flies positive for non-*Trypanozoon* trypanosomes (*T*. *vivax* n:11, *T*. *congolense s*.*l*. n:5, *T*. *grayi* n: 22, *T*. *simiae* n:2) which the LAMP kits did not cross-react with.

## Discussion

This study has demonstrated the potential of the Loopamp *Trypanosoma brucei* detection kit as a tool for identifying *Trypanozoon* DNA in tsetse. The LAMP kits showed a high degree of specificity, with no cross-reaction when challenged with non-target species of trypanosomes. LAMP was more sensitive than standard PCR in both laboratory-infected and wild-caught tsetse flies. Further we show that a single trypanosome-infected tsetse could be detected in a pool of 20 flies. Simplified DNA extractions were developed and the results show the ability to reduce the time of DNA extraction, although moderate equipment and skill is still required. The results suggest that LAMP may be a useful tool for epidemiological surveillance of *T*. *brucei s*.*l*. in HAT endemic regions to estimate the burden of the disease (in combination with species-specific PCR). This may be more cost-effective than active detection of human cases, and would allow control programmes to prioritise areas for HAT control.

The field extraction methods tested here have been assessed by balancing the length of the process, reliability at detecting positive samples and the number of steps involved in the method, which is a proxy for its complexity. The most successful method at identifying spiked samples was the ‘½ time Chelex method’ but once the complexity and time was taken into account ‘TE with Chelex’ was decided as the most efficient DNA extraction method. However, all of these extractions still require access to centrifuges, multiple reagents and handling steps and must be improved before implementation.

In previous studies, LAMP assays on pooled samples showed a decline in sensitivity by 60% with a ratio of 1:15 [18]. In the present study, the LAMP kit was capable of detecting one infected tsetse amongst 19 uninfected flies 100% of the time when coupled with the TE Chelex extraction method. This demonstrates that both the kit and the TE Chelex extraction have good potential for use as tools in the xenomonitoring of tsetse.

The serial dilution experiment confirms that the LAMP kits have an extremely high sensitivity in detecting trypanosomes. Previous studies have reported limits of detection ranging from the equivalent of 100 to 0.001 trypanosomes per ml [14, 15, 18] using in-house LAMP assays in accordance with results shown here. The advantage of using a commercially available, standardised LAMP kit would allow researchers to compare different settings with each other without differences in method confounding the results. The ability of the LAMP kits to detect such a low number of trypanosomes per ml is advantageous for its use in xenomonitoring as flies actively transmitting the disease can have infections ranging from <1 x 10^1^ to 1 x 10^4^ depending on the stage of infection[25]. However when viewing the results of the TBR and LAMP it is clear that the reliability of the two assays differ as although LAMP can detect lower concentrations of trypanosomes it also has a higher rate of false negatives at detectable levels of trypanosomes DNA. This could well be due to inhibitors in the DNA template and the nature of how the results are read out with the TBR results being run out on a gel in which exposure time can be adjusted to identify even very faint bands in the gel, whereas the LAMP results are viewed solely by eye and there is no means to identify low levels of amplification, even if it has occurred.

Another, less obvious, advantage with xenomonitoring is that the tsetse flies are continuously feeding on a wide range of local vertebrates[26] and could, in effect, act as an efficient source of blood for screening in order to detect *Trypanozoon* species in the environment. If tsetse are able to pick up *T*. *brucei s*.*l*. trypanosomes in blood meals and trypanosome DNA can be amplified, it would be possible to detect if HAT is present in a region. The results for *T*. *b*. *brucei* DNA persistence are highly encouraging as they demonstrate that it is possible to detect DNA from a blood meal 100% of the time after 48 hours post-feeding and in the extreme we have shown it is possible for the DNA to remain in a detectable state for up to six days in our colony flies. This is despite the fact that the tsetse flies in our experiment were not only digesting the initial infected blood meal, but they subsequently took on fresh blood, at later feeds, which helped flush their digestive system of previous meals.

The situation in the field may also be complicated by the presence of other trypanosome co-infections in the tsetse flies [27]. The results from the co-infection experiments demonstrate that even with co-infected flies it was still possible to identify those that had a *T*.*b*. *brucei* infection and in the one case where the fly cleared the *T*.*b*. *brucei* infection but maintained a *T*. *congolense* infection, the LAMP kit recorded this as a *T*.*b*. *brucei* negative fly.

The specificity of the LAMP kits was good when tested against single infections of *T*. *congolense* and *T*.*b*. *brucei*, with no cross-reaction with the flies infected with *T*. *congolense*.

In wild-caught flies, the LAMP test was more sensitive than the PCR based method, and was able to identify more *T*. *brucei s*.*l*. infections in the 449 flies caught from Uganda.

The LAMP kit used in the present study is only able to identify the *Trypanozoon* group, meaning that a species-specific PCR would still be necessary to differentiate human infective forms from the other trypanosomes in order to guide HAT control programmes effectively. Unfortunately the species-specific PCRs used to differentiate the *Trypanozoon* group use single-copy gene targets, making them significantly less sensitive than LAMP and other PCRs (e.g., TBR, ITS). In addition, it is currently not possible to conduct LAMP in a high-throughput manner, and the cost of kits is still significant, at $2.60 per test. A larger study involving cost analysis of PCR vs LAMP should be conducted to verify that molecular xenomonitoring is a suitable tool for studying the epidemiology of HAT and is able to highlight hotspot areas of transmission.

### Conclusion

This study has demonstrated a robust detection limit of the Loopamp *Trypanozoon* detection kit of 0.1 trypanosome per ml in dissected tsetse midguts. We demonstrated that the kit has high specificity, with no cross-reactivity in flies with multiple infections, including wild caught flies that had a greater variation of non-target species and provided a more realistic challenge. The kits could also detect *T*.*b*. *brucei* DNA 6 days after consumption of a contaminated blood meal, despite having fresh feeds every 48 hrs, which would be flushing their digestive system with clean blood. The sensitivity of the kits was high enough to allow for the detection of a single infected fly in a pool of 20 flies. The next step would be to test the DNA extraction and pooling methods in the field alongside a wider cost and feasibility evaluation of LAMP vs PCR and the ability to use xenomonitoring of HAT over time in the elimination campaign.
